# The ratio of TAPSE to PASP predicts prognosis in lung transplant candidates with pulmonary arterial hypertension

**DOI:** 10.1038/s41598-023-30924-1

**Published:** 2023-03-07

**Authors:** Satoshi Ishii, Shun Minatsuki, Masaru Hatano, Akihito Saito, Hiroki Yagi, Mai Shimbo, Katsura Soma, Takayuki Fujiwara, Hidetaka Itoh, Chihiro Konoeda, Masaaki Sato, Norifumi Takeda, Masao Daimon, Jun Nakajima, Issei Komuro

**Affiliations:** 1 Department of Cardiovascular Medicine, The University of Tokyo Hospital, 7-3-1 Hongo, Bunkyo-ku, Tokyo, 113-8655 Japan; 2 Department of Advanced Medical Center for Heart Failure, The University of Tokyo Hospital, 7-3-1 Hongo, Bunkyo-ku, Tokyo, 113-8655 Japan; 3 Department of Computational Diagnostic Radiology and Preventive Medicine, The University of Tokyo Hospital, 7-3-1 Hongo, Bunkyo-ku, Tokyo, 113-8655 Japan; 4 Department of Thoracic Surgery, The University of Tokyo Hospital, 7-3-1 Hongo, Bunkyo-ku, Tokyo, 113-8655 Japan; 5 Department of Clinical Laboratory, The University of Tokyo Hospital, 7-3-1 Hongo, Bunkyo-ku, Tokyo, 113-8655 Japan

**Keywords:** Cardiology, Cardiovascular diseases, Vascular diseases

## Abstract

Lung transplantation (LT) is the only option for patients with pulmonary arterial hypertension (PAH) refractory to maximal medical therapy. However, some patients referred for LT could survive without LT, and its determinants remain unclear. This study aimed to elucidate prognostic factors of severe PAH at the referral time. We retrospectively analyzed 34 patients referred for LT evaluation. The primary outcome was a composite of death or LT. Over a median follow-up period of 2.56 years, eight patients received LT and eight died. Compared with LT-free survival group, pulmonary arterial systolic pressure (PASP) was higher (*p* = 0.042), and the ratio of tricuspid annular plane systolic excursion (TAPSE) to PASP (TAPSE/PASP) was lower (*p* = 0.01) in LT or death group. In receiver operating characteristic analysis, the area under the curve was 0.759 (95% confidence interval 0.589–0.929) for TAPSE/PASP to predict primary outcome, and the optimal cut-off value was 0.30 mm/mmHg (sensitivity 0.875 and specificity 0.667). In a multivariate analysis, TAPSE/PASP was independently associated with death or LT. Kaplan–Meier analysis showed a better LT-free survival in patients with TAPSE/PASP ≧0.30 mm/mmHg than in those with < 0.30 mm/mmHg (*p* = 0.001). Low-level TAPSE/PASP could be a poor prognostic factor in PAH patients referred for LT evaluation.

## Introduction

Pulmonary arterial hypertension (PAH) is a progressive disorder characterized by elevated pulmonary arterial pressure and vascular resistance, eventually resulting in fatal respiratory and circulatory failure^1,2^. Recent therapeutic advances have improved the hemodynamics, symptoms, and prognosis of patients with PAH, and the number of patients referred for lung transplantation (LT) has decreased^3,4^; however, patients refractory to maximal targeted therapy for PAH have a high risk of death, and LT is the only curative treatment^5^.

Referral for LT evaluation is recommended when PAH patients prescribed optimal medical therapy show impaired functional status (The World Health Organization (WHO) functional class III or IV, six-minute walk distance < 350 m) with compromised hemodynamics (cardiac index < 2.0 L/min/m^2^, right atrial pressure > 15 mmHg), or signs of right heart failure^4–6^. However, the waiting period for LT in Japan is approximately three years due to the shortage of brain-dead donors, therefore PAH patients are often referred anticipating this period. It is substantially challenging even for transplant center specialists to accurately predict future clinical courses of referred PAH patients because some severe PAH patients deteriorate in an acute and rapid way, while the others survive for a long time under re-optimized medical therapy and eventually could pend LT even after listing for LT^7^. The difficulty in predicting a wide spectrum of clinical outcomes leads to variability among transplant centers regarding the referral and evaluation for LT^4^. Hence, there is an unmet need for establishing a straightforward prognostic clinical index for clinical predictions about patients with severe PAH, who are deteriorating under combination therapy.

In this retrospective study, we assessed the predictive value of clinical parameters obtained at the referral time for prognosis of severe PAH patients after referral to our transplant center, with the aim of developing a decision-making tool for LT candidates.

## Methods

### Study patients and data collection

**Figure 1:**
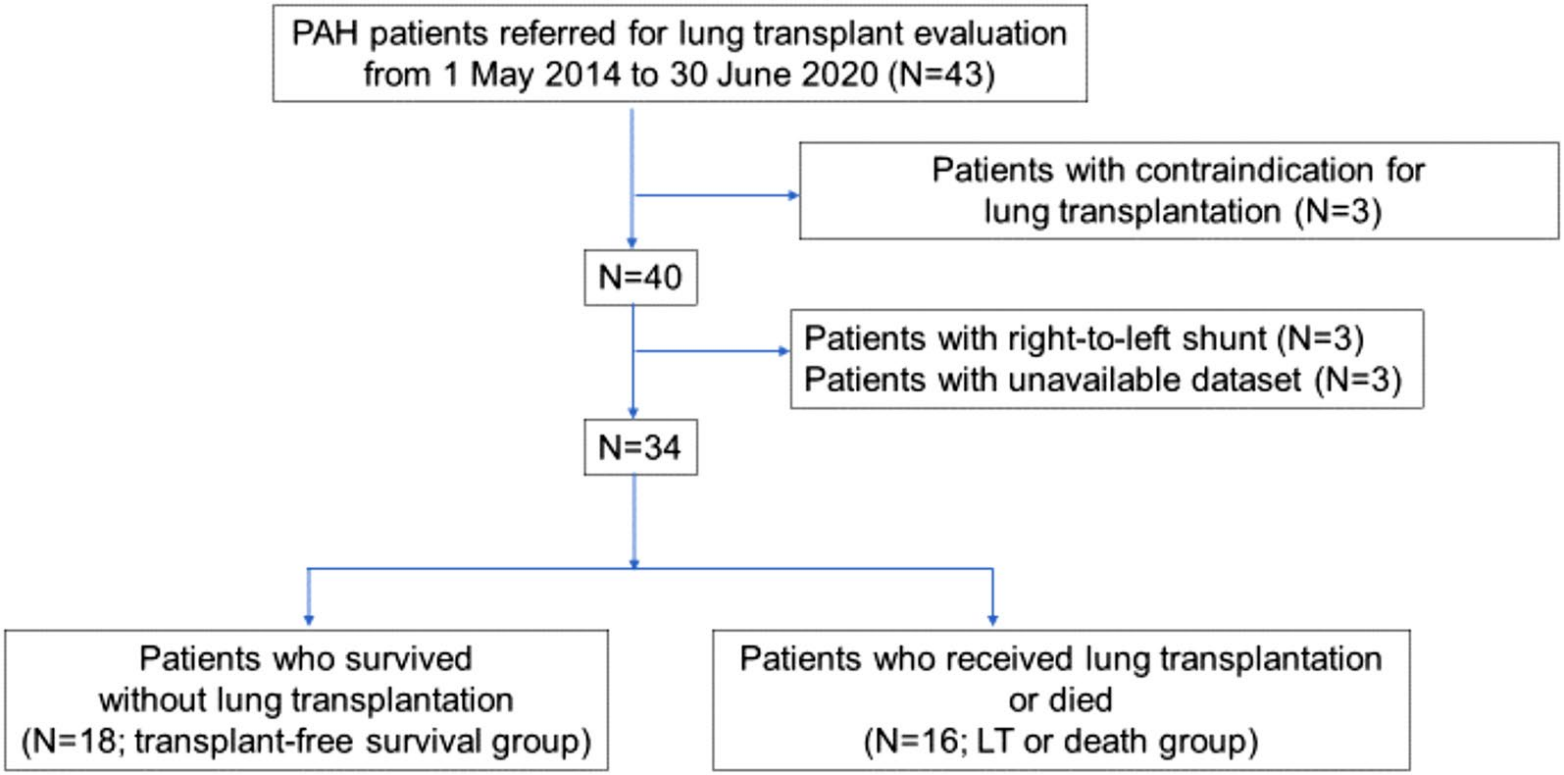
Flowchart of the study design, which included PAH patients referred for lung transplant evaluation. *LT* lung transplantation, *PAH* pulmonary arterial hypertension.

We conducted a retrospective cohort study by analyzing the records of consecutive 43 patients with PAH referred for LT evaluation at the University of Tokyo Hospital from May 2014 to June 2020 (Fig. 1). The patients were in WHO functional class III or IV and had compromised hemodynamics despite optimal medical therapy. Among the 43 patients, three with contraindications to LT (hypertrophic cardiomyopathy, malignancy, or lack of family support) were excluded. Furthermore, three patients with right-to-left intracardiac shunt (one with patent ductus arteriosus and two with Eisenmenger’s syndrome due to atrial septal defect or ventricular septal defect) were also excluded from this study, as previously reported^8^. Among the remaining of 37 patients, three patients with unavailable echocardiographic data were excluded, and 34 patients whose functional, laboratory, echocardiographic, and hemodynamic data was available were included in the analysis. The data for analysis was at the time of referral. The present study was performed according to the ethical guidelines of the University of Tokyo (approval by the Ethical Committee of the University of Tokyo: No. 2650) and in accordance with the Declaration of Helsinki. Due to nature of retrospective study, written informed consent was waived according to the approval by the Ethical Committee of the University of Tokyo.

### Hemodynamic measurements

Hemodynamic parameters were measured in the cardiac catheterization laboratory during right heart catheterization. Cardiac output was measured by the Fick method.

### Echocardiographic measurements

Participants underwent 2-dimensional echocardiography performed by experienced operators using commercially available devices in accordance with the guidelines of the American Society of Echocardiography^9^. Semi-quantitative assessment of tricuspid regurgitation (TR) was performed according to the guidelines of the American Society of Echocardiography and the severity of TR was classified into four stages: none, mild, moderate, or severe^10^. Right ventricle (RV) systolic function was evaluated by RV fractional area change (FAC), tricuspid annular plane systolic excursion (TAPSE), and tissue Doppler–derived tricuspid lateral annular systolic velocity. FAC was calculated from the RV-focused apical four-chamber view by the following formula: FAC (%) = (RV end diastolic area − RV end systolic area)/RV end diastolic area × 100. TAPSE was measured on the M-mode tracing obtained from the RV-focused apical four-chamber view using the distance of systolic excursion of the RV annular segment along its longitudinal plane. Tissue Doppler–derived tricuspid lateral annular systolic velocity was measured as a peak systolic wave on tricuspid lateral annulus from the RV-focused apical four-chamber view using tissue Doppler imaging. TR pressure gradient was calculated from the continuous-wave Doppler TR velocity by the simplified Bernoulli equation. Pulmonary artery systolic pressure (PASP) was calculated by the formula PASP = 4 × (peak velocity of TR)^2^ + estimated right atrial pressure. Right atrial pressure was based on inferior vena cava diameter and collapsibility, following the guidelines^11^. In addition, we calculated TAPSE/PASP as load-independent parameters for assessing RV function because RV function is sensitive to change in afterload, known as RV-pulmonary circulation (PC) coupling^12^.

### Management of LT waiting patients

After LT evaluation, patients were registered with the Japan Organ Transplantation Network, where the waitlist order system is adopted for allocating cadaveric lungs. Our advanced PAH therapeutics team including cardiologists, respiratory surgeons, nurses, and transplant coordinators regularly reviewed the clinical state of the listed patients, and determined whether the patients should receive LT on their turn with deteriorating signs of right heart failure and hemodynamics, or pend LT and continue medication therapy on clinically stable conditions that lowering of mean pulmonary arterial pressure (mPAP), which is the most important prognostic determinant in Japan^6^, is achieved without worsening right heart failure.

### Outcome

Patients were followed up from the date of referral for LT evaluation to August 31, 2021. The primary outcome was a composite of LT or death, as in previous reports^13–15^. LT was included as a primary outcome because LT was considered as last resort treatment option for severely ill PAH patients at higher risk of death under maximal medical therapy and as a treatment for patients who are at the limitation of medical therapy. Patients who survived without LT were censored on August 31, 2021.

### Statistical analyses

All statistical analyses were performed using SPSS statistics 19 (SPSS Inc, Chicago, IL, USA). All data are expressed as mean ± standard deviation or median (interquartile range) unless otherwise specified. Continuous variables between groups were compared using unpaired t-test for normally distributed data or Mann–Whitney test for non-normally distributed data. Categorical variables were compared using Fisher’s exact test. Correlation between two variables were evaluated by Spearman method. Univariate Cox proportional hazards analysis was used to assess factors associated with death or need for LT. Age, sex and significant parameters on univariate Cox proportional hazards analysis were subsequently included in the multivariate Cox proportional hazard models. Receiver operating characteristic analysis was performed to assess the discriminatory ability of the parameters in the prediction of death or need for LT, and the optimal cut-off value was obtained from receiver operating characteristic analysis using Youden’s index. Kaplan–Meier analysis was performed to assess the differences in LT-free survival among subjects according to the dichotomous classification of variables, and the log-rank test was used to compare the distribution of LT-free survival. For all analyses, *p* value < 0.05 was considered statistically significant.

## Results

### Baseline characteristics

In total, 34 potential transplant candidates with severe PAH were included in this analysis. The mean age was 29.0 ± 10.3 years old, and 24 (71%) were female. During a median follow-up of 2.56 years (1.63–3.11 years), eight patients underwent LT and eight died of severe respiratory and circulatory failure. The 34 patients were categorized into two groups: LT or death group (n = 16) and LT-free survival group (n = 18). Table 1 summarizes the patients’ demographic, functional, laboratory, echocardiographic and hemodynamic characteristics at the time of referral for LT evaluation. The hemodynamic and echocardiographic measures were done at the median interval of 9.5 days (4.0–43.0 days). There were no differences in age, sex, forms of PAH, prescribed medicine use or WHO functional class between two groups. Most patients were diagnosed with idiopathic or heritable PAH and received PAH-specific combination therapy including parental prostanoid analogs. They were classified as WHO functional class III or IV, and there was no difference in six-minute walk distance between groups.

**Table 1:** Baseline characteristics of PAH patients referred for lung transplant evaluation.

	Transplant-free survival (N = 18)	LT or death (N = 16)	*p* Value
Observational period (years)	2.92 (1.84–4.19)	1.96 (0.72–2.61)	0.028
Age (years)	27.5 ± 9.2	30.8 ± 11.4	0.366
Sex (male)	4 (22.2%)	6 (37.5%)	0.457
BMI (kg/m^2^)	19.4 ± 2.6	19.6 ± 2.1	0.837
Diagnosis, n (%)			0.648
I/H PAH	16 (88.9%)	13 (81.2%)	
CTD-PAH	2 (11.1%)	3 (18.8%)	
Medical history			
Hypertension	0	0	
Diabetes	0	0	
Smoking	3 (16.7%)	5 (31.3%)	0.429
Medication management, n (%)			
Epoprostenol	13 (72.2%)	11 (68.8%)	1
Treprostinil	4 (22.2%)	4 (25.0%)	1
Selexipag	2 (11.1%)	1 (6.3%)	1
PDE-V inhibitor	11 (61.1%)	10 (62.5%)	1
sGC stimulator	7 (38.9%)	5 (31.3%)	0.729
ERA	17 (94.4%)	16 (100%)	1
Combination therapy, n (%)			1
Two	2 (11.1%)	2 (12.5%)	
More than three	16 (88.9%)	14 (87.5%)	
WHO functional class, n (%)			0.457
III	18 (100%)	14 (87.5%)	
IV	0	2 (12.5%)	
Six-minute walk distance (m)	421.2 ± 58.9	392.3 ± 186.7	0.559
Laboratory data			
Hemoglobin (g/dL)	12.4 ± 1.6	12.0 ± 1.7	0.505
Albumin (g/dL)	4.1 ± 0.3	4.1 ± 0.5	0.878
ALT (U/L)	14.5 (10–20)	11.0 (7–14.5)	0.238
BUN (mg/dL)	11.6 (10.0–14.2)	13.0 (11.1–16.5)	0.133
Echocardiography			
RVFAC (%)	30.8 ± 6.0	29.8 ± 11.6	0.748
TR severity, n (%)			0.039
≦ Moderate	18 (100%)	12 (75.0%)	
Severe	0	4 (25.0%)	
RV S’ (cm/s)	12.4 ± 1.7	12.9 ± 2.9	0.539
PASP (mmHg)	66.4 ± 15.6	81.5 ± 25.4	0.042
TAPSE (mm)	21.1 ± 4.5	18.1 ± 3.9	0.053
TAPSE/ PASP (mm/ mmHg)	0.32 (0.26–0.39)	0.24 (0.18–0.29)	0.01
Right heart catheterization			
mRAP (mmHg)	8 (7–9)	6.5 (5–9.5)	0.465
mPAP (mmHg)	45 (36–67)	54 (48–58)	0.277
mPCWP (mmHg)	11 (9–13)	10 (7–11)	0.078
Cardiac Index (L/min/m^2^)	3.86 ± 1.00	3.41 ± 1.17	0.241
PVR (wood unit)	7.4 ± 3.7	9.4 ± 3.7	0.13
SvO_2_ (%) (n = 27)	72.3 ± 1.4 (n = 15)	66.3 ± 3.0 (n = 12)	0.008

*ALT* alanine aminotransferase, *BMI* body mass index, *BUN* blood urea nitrogen, *CTD-PAH* connective tissue disease associated pulmonary arterial hypertension, *ERA* endothelin receptor antagonist, *I/H PAH* idiopathic/hereditary pulmonary arterial hypertension, *mPAP* mean pulmonary arterial pressure, *mPCWP* mean pulmonary capillary wedge pressure, *mRAP* mean right atrial pressure, *PASP* pulmonary arterial systolic pressure, *PVR* pulmonary vascular resistance, *RVFAC* right ventricular fractional area change, *RV S’* systolic annular tissue velocity of the lateral tricuspid annulus, *sGC* soluble guanylate cyclase stimulator, *SvO*_*2*_ mixed venous oxygen saturation, *TAPSE* tricuspid annular plane systolic excursion, *TR* tricuspid regurgitation, *WHO* World Health Organization.

Hemodynamic studies showed that both groups presented with severe pre-capillary pulmonary hypertension under PAH combination therapy. The LT or death group showed higher mPAP, lower cardiac index, and a more elevated pulmonary vascular resistance, albeit not statistically significant. Among echocardiographic measurements, RVFAC decreased across the groups. The LT or death group tended to have lower TAPSE and significantly higher PASP with higher severity of TR. Furthermore, TAPSE/PASP ratio, a non-invasive index of the RV to PC coupling state, was significantly lower in the LT or death group than the LT-free survival group. There was no significant correlation between TAPSE/PASP and mean right atrial pressure (r =  − 0.289, *p* = 0.097). On the other hand, TAPSE/PASP and pulmonary vascular resistance were significantly correlated with each other (r =  − 0.544, *p* = 0.001).

### Prognostic significance of TAPSE/PASP

**Figure 2:**
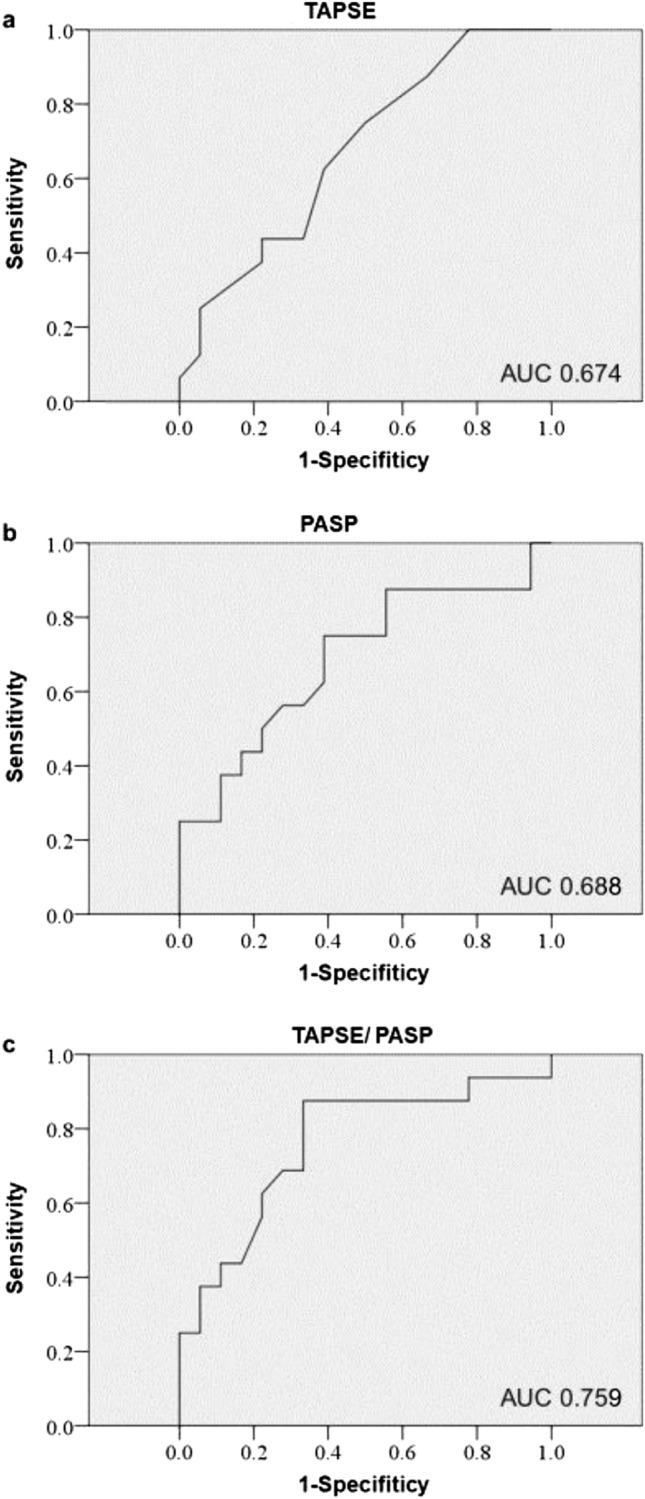
Receiver operating characteristic analysis on TAPSE, PASP and TAPSE/PASP at the referral time for prediction of death or need for LT. *AUC* area under the curve, *LT* lung transplantation, *PASP* pulmonary arterial systolic pressure, *TAPSE* tricuspid annular plane systolic excursion.

The result of univariable Cox regression analysis of factors associated with LT or death after referral for LT evaluation are summarized in Table 2. WHO functional class, PASP, and TAPSE/PASP at the time of referral were significantly associated with death or LT. Receiver operating characteristic analysis of PASP, TAPSE, and TAPSE/PASP for the prediction of death or LT showed that TAPSE/PASP had the largest area under the curve at 0.759 (95% confidence interval 0.589–0.929), compared with that of PASP (0.688, 95% confidence interval 0.504–0.871) and that of TAPSE (0.674, 95% confidence interval 0.493–0.854) (Fig. 2). TAPSE/PASP had a highly sensitive and specific predictive value of death or need for LT with the optimal cut-off of 0.30 mm/mmHg based on Youden’s index (sensitivity: 0.875, and specificity 0.667).

**Table 2:** Univariate and multivariate Cox proportional analysis of factors associated with LT or death after referral for LT evaluation.

Parameter	Univariate		Multivariate	
	Hazard ratio (95% CI)	*p* Value	Hazard ratio (95% CI)	*p* Value
Sex (male)	1.628 (0.588–4.502)	0.348	3.032 (0.913–10.072)	0.07
Age (years)	1.029 (0.978–1.084)	0.269	1.026 (0.966–1.090)	0.41
WHO functional class	17.777 (2.925–108.025)	0.002	5.616 (0.554–56.933)	0.144
Six-minute walk distance (m)	0.995 (0.990–1.000)	0.063		
mRAP (mmHg)	1.075 (0.893–1.295)	0.446		
mPAP (mmHg)	1.025 (0.990–1.061)	0.167		
PVR (wood unit)	1.123 (0.992–1.273)	0.068		
mPCWP (mmHg)	0.856 (0.689–1.063)	0.159		
Cardiac index (L/min/m^2^)	0.653 (0.372–1.147)	0.138		
RVFAC (%)	1.006 (0.946–1.071)	0.838		
RV S’ (cm/ s)	1.098 (0.869–1.387)	0.434		
TAPSE (mm)	0.905 (0.807–1.016)	0.091		
PASP (mmHg)	1.045 (1.018–1.073)	0.001		
TAPSE/PASP (mm/mmHg)	0.925 (0.871–0.982)	0.011	0.917 (0.855–0.984)	0.016

*CI* confidence interval, *mPAP* mean pulmonary arterial pressure, *mPCWP* mean pulmonary capillary wedge pressure, *mRAP* mean right atrial pressure, *PASP* pulmonary arterial systolic pressure, *PVR* pulmonary vascular resistance, *RVFAC* right ventricular fractional area change, *RV S’* systolic annular tissue velocity of the lateral tricuspid annulus, *TAPSE* tricuspid annular plane systolic excursion.

In multivariate Cox regression analysis, PASP was discarded to avoid collinearity, and TAPSE/PASP was significantly and independently associated with death or LT after adjusting for age, sex and WHO functional class.

### Survival analysis

**Figure 3:**
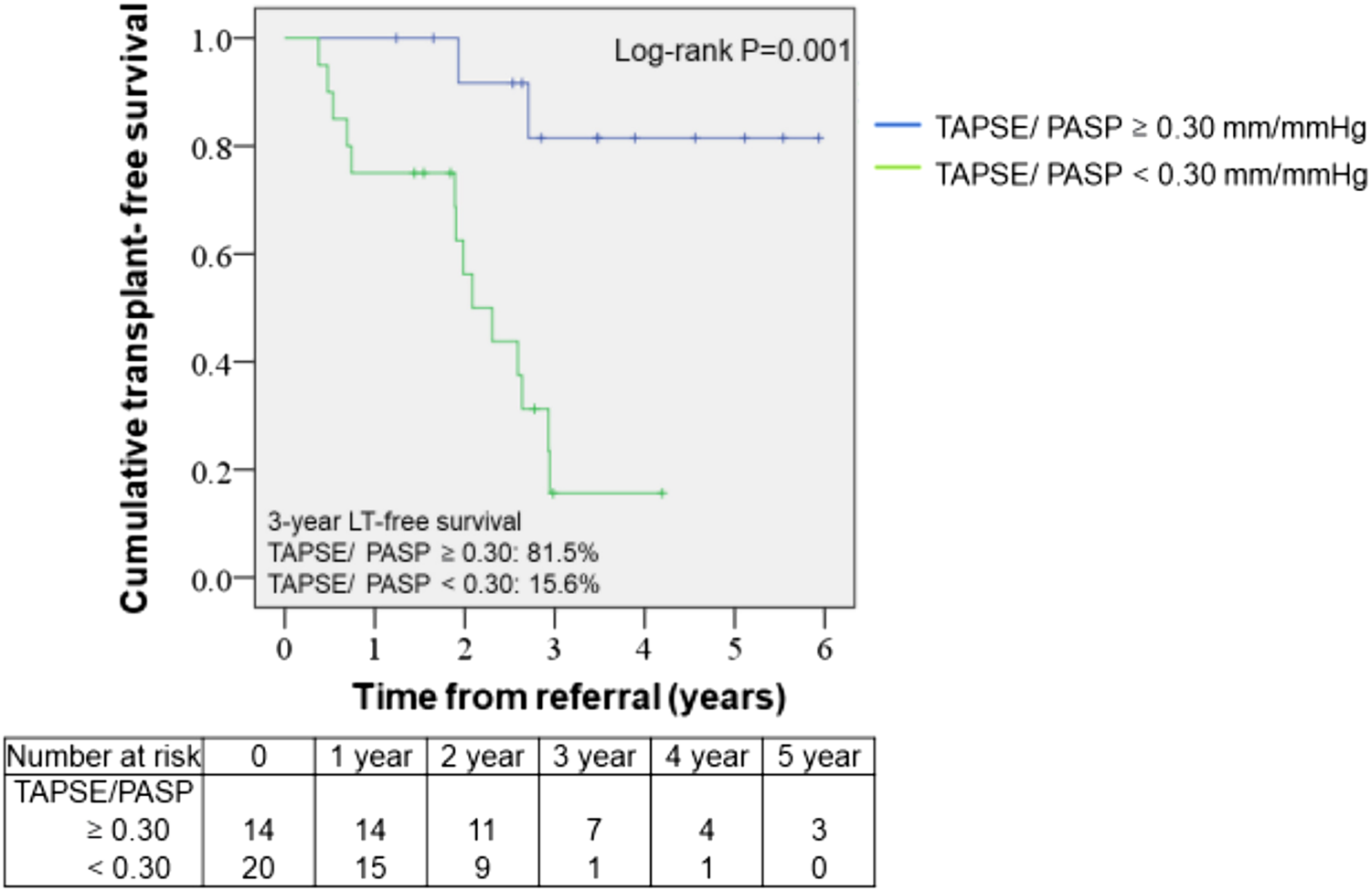
Kaplan–Meyer analysis for freedom from death or LT after referral for LT. PAH patients are stratified according to TAPSE/PASP level at the referral time into two groups (TAPSE/PASP ≧ 0.30, n = 14; TAPSE/PASP < 0.30, n = 20). *LT* lung transplantation, *PAH* pulmonary arterial hypertension, *PASP* pulmonary arterial systolic pressure, *TAPSE* tricuspid annular plane systolic excursion.

Based on our results, we further performed Kaplan–Meier survival analysis for freedom from death or LT using a TAPSE/PASP cut-off value of 0.30 mm/mmHg (Fig. 3). There were 14 patients with TAPSE/PASP ≧ 0.30 mm/mmHg and 20 patients with TAPSE/PASP < 0.30 mm/mmHg. Patients with TAPSE/PASP < 0.30 mm/mmHg had significantly worse LT-free survival (log-rank test, *p* = 0.001); LT-free survival rates for the first, second, and third-years were 75.0%, 56.2%, and 15.6%, respectively. On the other hand, patients with TAPSE/PASP ≧ 0.30 mm/mmHg presented with a better LT-free survival and LT-free survival rates for the first, second, and third-years were 100%, 91.7% and 81.5%, respectively.

## Discussion

This study investigated the prognostic significance of demographic, functional, echocardiographic, and hemodynamic parameters at the time of referral to our transplant center in potential LT candidates with severe PAH. We demonstrated that the echocardiographic index of TAPSE/PASP, a non-invasive surrogate of RV-PC coupling, was lower in patients who had undergone LT or died during the follow-up period, and was significantly associated with prognosis after referral with a cut-off value of 0.30 mm/mmHg.

RV function plays a key role in determining symptoms and survival in severe PAH patients^16^. It is affected by the vascular load of PC, and a declining RV function with progressive uncoupling to the PC is a turning point of clinical worsening in PAH^12^. PAH patients should be referred to a transplant center for LT evaluation when they show signs of right heart failure despite the maximum medical therapies^3^, and thus it is important to recognize changes in patient condition on RV-PC coupling. The gold standard for evaluating RV-PC coupling is the ratio of ventricular elastance to arterial elastance, which is measured using invasive pressure–volume loop. However, this method is technically difficult to practically use in daily medical care^12^, and surrogates of the ratio of ventricular elastance to arterial elastance using echocardiography or magnetic resonance imaging resonance have been actively explored^17^. TAPSE/PASP is a recently proposed non-invasive echocardiographic index, which has a prognostic value not only in patients with left heart failure^18–21^, but also in patients with PAH^22^. Tello et al.^23^ reported that, among five surrogates for RV-PC coupling including TAPSE/PASP, FAC/mPAP, and RV area change/end-systolic area, TAPSE/PASP is the most valuable predictor of the ratio of ventricular elastance to arterial elastance in patients with severe pulmonary hypertension whose mPAP and pulmonary vascular resistance were 47 mmHg and 6.9 Wood Units, respectively. However, it remains unknown whether assessment of RV-PC coupling using TAPSE/PASP predicts the outcome of patients with severe PAH referred to a transplant center for LT evaluation. In our cohort composed of potential LT candidates, TAPSE/PASP, which inversely correlated with pulmonary vascular resistance as previously reported^22^, was significantly lower in the LT or death group than in the LT-free survival group, indicating more impaired RV-PC coupling and decreased RV functional reserve capacity at referral in the LT or death group. Furthermore, multivariate Cox analysis showed that TAPSE/PASP at referral was strongly and independently associated with worse prognosis among various clinical parameters. These results suggest that TAPSE/PASP at referral time could reflect the stages of RV-PC coupling in potential LT candidates and could predict clinical outcomes after referral.

In the present study, patients with TAPSE/PASP < 0.30 mm/mmHg were more likely to undergo LT or die, whereas those with TAPSE/PASP ≧ 0.30 mm/mmHg had a better LT-free survival even after referral for LT evaluation. In the aforementioned study by Tello et al.^23^, severe pulmonary hypertension patients with TAPSE/PASP < 0.31 mm/mmHg had a significantly worse prognosis than those with higher TAPSE/PASP. In our study cohorts with more impaired functional and hemodynamics status despite higher prevalence of combination therapies including parental prostanoid analogue, receiver operating characteristic analysis showed that the optimal TAPSE/PASP cut-off for death or need for LT was 0.30 mm/mmHg. This result indicates that also in Japan, with the circumstances of lung transplantation varying from other countries, TAPSE/PASP at referral for LT evaluation would be useful to stratify subsequent clinical progression and outcome into two groups: patients with impaired RV-PC coupling (TAPSE/PASP < 0.30 mm/mmHg) are at high risk of death or undergoing LT, and those with preserved RV-PC coupling (TAPSE/PASP ≧ 0.30 mm/mmHg) are likely to survive without undergoing LT under re-optimized medical therapy.

In the present analysis, previously reported prognostic factors in PAH at referral time, including six-minute walk distance^24^, TAPSE^25^ or invasively measured hemodynamic parameters such as right atrial pressure, cardiac index, mPAP or pulmonary vascular resistance^15,26^, were not associated with worse clinical outcomes after referral. This might be because of differences in study design, sample size, or cohort background. Our finding that PAH patients with TAPSE/PASP ≧ 0.30 mm/mmHg had a better LT-free survival even after referral for LT corresponds to the latest risk assessment in PAH, in which TAPSE/PASP criteria is newly added with the cut-off value of 0.32 mm/mmHg to determine low-risk status^27^. However, because the current concept of risk stratification in PAH consists of multi-parameter measurements including functional, biomarker, non-invasive imaging, and invasive hemodynamics studies and mPAP is adopted as the most important prognostic determinant in Japan^6,27^, further investigation on larger cohorts is strongly warranted to confirm whether TAPSE/PASP can act as a surrogate of RV-PC coupling and add a practical value in stratifying patients with severe PAH referred for LT evaluation.

Our study had several limitations. First, it was a single-center retrospective study with a small sample size in Japan, where the lung allocation score and high-priority list have not yet been adopted. Future research with a larger sample size is needed. Second, we did not evaluate subsequent echocardiographic changes. Although we conducted a multivariate analysis, there could be unmeasured confounders and residual bias, and previously reported prognostic parameters such as right atrial size and RV basal diameter could not be used. Third, there could be intra- and inter-observer variabilities of echocardiographic measurements, including TAPSE and PASP. Fourth, TAPSE shows no further decrease once it reaches the lowest threshold. Further research is needed to confirm the utility of the TAPSE/PASP ratio in PAH patients with severe right heart failure. Fifth, we could not evaluate cardiac magnetic resonance imaging, which is the gold standard for assessing RV function. However, echocardiography is a reliable modality with lower cost and more accessibility and can be performed safely for patients with severe PAH receiving parental prostanoid analogs.

## Conclusions

In conclusion, low-level TAPSE/PASP ratio was significantly associated with death or LT in PAH patients referred for LT, suggesting the potential clinical importance of echocardiographic assessment of TAPSE/PASP changes while optimizing medical therapy, and the cut-off value of 0.30 mm/mmHg predicted their future clinical courses.

## Data Availability

The datasets generated during and/or analyzed during the current study are available from the corresponding author on reasonable request.
